# Construction and evaluation of endometriosis diagnostic prediction model and immune infiltration based on efferocytosis-related genes

**DOI:** 10.3389/fmolb.2023.1298457

**Published:** 2024-01-04

**Authors:** Fang-Li Pei, Jin-Jin Jia, Shu-Hong Lin, Xiao-Xin Chen, Li-Zheng Wu, Zeng-Xian Lin, Bo-Wen Sun, Cheng Zeng

**Affiliations:** ^1^ The First Affiliated Hospital of Guangzhou University of Chinese Medicine, Guangzhou, China; ^2^ Guangzhou University of Chinese Medicine, Guangzhou, China

**Keywords:** endometriosis, efferocytosis, immune infiltration, bioinformatics, and machine learning

## Abstract

**Background:** Endometriosis (EM) is a long-lasting inflammatory disease that is difficult to treat and prevent. Existing research indicates the significance of immune infiltration in the progression of EM. Efferocytosis has an important immunomodulatory function. However, research on the identification and clinical significance of efferocytosis-related genes (EFRGs) in EM is sparse.

**Methods:** The EFRDEGs (differentially expressed efferocytosis-related genes) linked to datasets associated with endometriosis were thoroughly examined utilizing the Gene Expression Omnibus (GEO) and GeneCards databases. The construction of the protein-protein interaction (PPI) and transcription factor (TF) regulatory network of EFRDEGs ensued. Subsequently, machine learning techniques including Univariate logistic regression, LASSO, and SVM classification were applied to filter and pinpoint diagnostic biomarkers. To establish and assess the diagnostic model, ROC analysis, multivariate regression analysis, nomogram, and calibration curve were employed. The CIBERSORT algorithm and single-cell RNA sequencing (scRNA-seq) were employed to explore immune cell infiltration, while the Comparative Toxicogenomics Database (CTD) was utilized for the identification of potential therapeutic drugs for endometriosis. Finally, immunohistochemistry (IHC) and reverse transcription quantitative polymerase chain reaction (RT-qPCR) were utilized to quantify the expression levels of biomarkers in clinical samples of endometriosis.

**Results:** Our findings revealed 13 EFRDEGs associated with EM, and the LASSO and SVM regression model identified six hub genes (ARG2, GAS6, C3, PROS1, CLU, and FGL2). Among these, ARG2, GAS6, and C3 were confirmed as diagnostic biomarkers through multivariate logistic regression analysis. The ROC curve analysis of GSE37837 (AUC = 0.627) and GSE6374 (AUC = 0.635), along with calibration and DCA curve assessments, demonstrated that the nomogram built on these three biomarkers exhibited a commendable predictive capacity for the disease. Notably, the ratio of nine immune cell types exhibited significant differences between eutopic and ectopic endometrial samples, with scRNA-seq highlighting M0 Macrophages, Fibroblasts, and CD8 Tex cells as the cell populations undergoing the most substantial changes in the three biomarkers. Additionally, our study predicted seven potential medications for EM. Finally, the expression levels of the three biomarkers in clinical samples were validated through RT-qPCR and IHC, consistently aligning with the results obtained from the public database.

**Conclusion:** we identified three biomarkers and constructed a diagnostic model for EM in this study, these findings provide valuable insights for subsequent mechanistic research and clinical applications in the field of endometriosis.

## 1 Introduction

Endometriosis (EM) is a long-lasting inflammatory condition reliant on estrogen, impacting around 10% of women globally during their reproductive years (Sun et al., 2022). The pathology of EM is marked by the emergence and proliferation of the endometrium outside the uterine cavity, with infiltration and recurrent bleeding, resulting in a local chronic inflammatory response (Ruiz-Alonso et al., 2017). EM then progresses to nodules or masses, pain, and infertility. Nevertheless, the advancement of the condition tends to be gradual, often requiring 7–10 years for noticeable clinical symptoms to manifest. This prolonged timeline contributes to delays in both diagnosing and administering optimal treatment for EM (Bakhtiarizadeh et al., 2018). The pathogenesis of EM has not been clarified, and the most controversial explanation is based on the theory of menstrual reflux proposed by Sampson in 1921. While 90% of women undergo menstrual reflux, only 10% contend with EM, indicating that the disease involves a more intricate pathogenesis (He et al., 2023). Recently, an increasing body of research has emphasized the significance of immune irregularities in the development of EM. For instance, studies have revealed immune cell infiltration at ectopic lesions and elevated production of pro-inflammatory cytokines and chemokines (Matarese et al., 2003; Meng-Hsing et al., 2005; Lauren et al., 2009; Capobianco and Rovere-Querini, 2013; Ahn et al., 2016). Therefore, it is necessary to further clarification of the regulatory mechanisms of immune infiltration in EM and screen more accurate specific biomarkers for EM. These biomarkers can also be used for the early diagnosis and effective therapeutic targets for EM.

Efferocytosis plays an essential role in immunomodulation and serves as a central mechanism in removing aberrant cells, pathogens, and cellular debris (Morioka et al., 2019). Efferocytosis is a consecutive process involving phagocyte recognition, phagocytosis, and the subsequent breakdown of apoptotic cells. This intricate process is intricately governed by a dynamic interplay of molecular signaling and cellular receptors. This contributes to the efficient and effective removal of apoptotic cells while minimizing the potential for autoimmune reactions (Doran et al., 2020). Efficient efferocytosis prevents the occurrence of secondary necrosis and the shed of inflammatory factors and poisonous molecules from deceased cells, thus, it is crucial for maintaining tissue homeostasis and promoting inflammation repair or injury response (Doran et al., 2020). Recent studies have suggested that efferocytosis has a profound impact on the inflammatory microenvironment and that defective efferocytosis represents a key mechanism driving the occurrence and progression of chronic phlogistic diseases, comprising atherosclerosis, systemic lupus erythematosus, cancer, and chronic obstructive pulmonary disease (Asseldonk et al., 2008; Tajbakhsh et al., 2021; Doddapattar et al., 2022; Zhang et al., 2022).

Early detection and prevention in the clinical management of EM are challenging. Due to the disease being characterized by local chronic inflammation and immune infiltration, the mechanisms underlying such phenomenon might be explained from the perspective of efferocytosis. Earlier investigations have unveiled connections between genes linked to ferroptosis, cuproptosis, and autophagy, and the identification of EM (Li et al., 2021; Ji et al., 2022), but the association between efferocytosis and EM has fewer reported. Bioinformatics can identify potential new biomarkers of disease and their diagnostic role, it represents an emerging technology in the current biomedical field, which uses modern information tools (Jäger, 2022; Ji et al., 2022; Wei et al., 2023). Hence, conducting a thorough analysis of the regulatory functions of pivotal genes of efferocytosis and the correlation with immune infiltration in EM using bioinformatics and machine learning techniques to represent a novel direction for the diagnosis, prophylaxis, and therapy of EM patients. In addition, our findings were validated by RT-qPCR and IHC analyses of real-world EM tissues.

## 2 Material and methods

### 2.1 Data source and preprocessing

#### 2.1.1 Microarray data

The Gene Expression Omnibus (GEO) database (https://www.ncbi.nlm.nih.gov/geo) contains genetic data related to various physiological and pathological states uploaded by research sites worldwide. To obtain gene expression matrix data related to EM, we searched the database using the following terms: “endometriosis” [Title] AND “ *Homo sapiens*” [porgn] AND “expression profiling by array” [Filter]. The following samples were included: mRNA expression profiles obtained by high-throughput sequencing, and the selected datasets to include both ovarian endometriosis lesions (ectopic endometria) and eutopic endometria. Eutopic and ectopic endometrial samples were procured from identical patients, with the eutopic endometria serving as the control for the corresponding ectopic endometrial sample in each patient. The outcomes of the inquiry were verified by two researchers before inclusion in the study.

We retrieved GSE7305 (GPL570, Affymetrix Human Genome U133 Plus 2.0 Array) (Hever et al., 2007), GSE11619 (GPL96, Affymetrix Human Genome U133 Plus 2.0 Array) (Hull et al., 2008), and GSE25628 (GPL571, Affymetrix Human Genome U133 Plus 2.0 Array) (Crispi et al., 2013) datasets from the GEO database. The GSE7305 dataset comprised 10 pairs of endometrial samples, the GSE11619 dataset encompassed 9 pairs of endometrial samples, and the GSE25628 dataset involved 8 pairs of endometrial samples. The original “CEL” files for the mentioned datasets were acquired from the GEO database. as the training cohort, which was adjusted for the background and normalized with “affy” and “simpleaffy” R packages (Gautier et al., 2004; Wilson and Miller, 2005). Based on the probe annotation file for each dataset, the probes were converted into gene symbols. Probes lacking expression values were excluded, and for those instances where different probes mapped to the same gene, the mean expression value was computed. To mitigate batch effects, the “sva” R package was utilized (Leek et al., 2012). Moreover, principal component analysis (PCA) was employed to appraise the distribution patterns among EM and normal samples in the microarray datasets. GSE37837 (GPL6480, Agilent-014850 Whole Human Genome Microarray 4 × 44 K G4112F) (Khan et al., 2012) contained 8 pairs of endometria and GSE6364 (GPL570 Affymetrix Human Genome U133 Plus 2.0 Array) (Burney et al., 2007) contained 16 normal endometria and 19 ectopic endometria were used as the test cohorts. Agilent data were normalized using the “normalizeBetweenArrays” and “backgroundCorrect” functions in the “limma” R package (Ritchie et al., 2015), and the duplicate probes associated with the same gene in each dataset were averaged using the “avereps” function within the “limma” R package. The details of the endometriosis-related gene datasets downloaded from the GEO database are presented in Table 1.

**TABLE 1:** Summary of the primary studies encompassed in the integrated analysis of genome-wide expression studies for EM.

Dataset	GEO accession No	Sample size	Eutopic	Ectopic	Platform
Training set (*n* = 54)	GSE7305	20	10 (tissues adjacent to EMs)	10	GPL570 [HG-U133_Plus_2] Affymetrix Human Genome U133 Plus 2.0 Array
	GSE11691	18	9 (tissues adjacent to EMs)	9	GPL96 [HG-U133A] Affymetrix Human Genome U133A Array [HG-U133A_2] Affymetrix Human Genome U133A 2.0 Array
	GSE25628	16	8 (tissues adjacent to EMs)	8	GPL96 [HG-U133A] Affymetrix Human Genome U133A Array
Test set 1 (*n* = 16)	GSE37837	16	8 (tissues adjacent to EMs)	8	Agilent-014850 Whole Human Genome Microarray 4 × 44 K G4112F (Probe Name version)
Test set 2 (*n* = 35)	GSE6364	35	16 (tissues from healthy people)	19	GPL570 [HG-U133_Plus_2] Affymetrix Human Genome U133 Plus 2.0 Array

#### 2.1.2 Selection of efferocytosis-related genes

GeneCards databases (https://www.genecards.org/) is an extensive bioinformatics database offering concise information on genomes, proteomes, and associated human genetic data. The Kyoto Encyclopedia of Genes and Genomes (KEGG) database is a comprehensive biological information database. In addition to the KEGG pathway, the database also contains data from many other modules, including genome information. The keyword “Efferocytosis” was used to select the efferocytosis-related genes (EFRGs) from GeneCards databases and the KEGG databases.

### 2.2 Selection of differentially expressed efferocytosis-related genes

The “limma” R package was employed to filter the differentially expressed genes (DEGs) between eutopic and ectopic endometrial specimens in the processed training set (Leek et al., 2012). The criteria for the output included absolute values of the |log2-FC|> 1 and a *p* < 0.05. Here, logFC >0 and logFC <0 denoted upregulated and downregulated genes, respectively. Volcano plots and heatmaps illustrating the DEGs were created using the “ggplot2” and “heatmap” R packages. Then, we identified differentially expressed efferocytosis-related genes (EFRDEGs) via the “VennDiagram” R package.

### 2.3 Construction of EFRDEGs PPI and TF network

The STRING database (https://string-db.org/) is an online resource for analyzing protein-protein interactions. To reveal the interactions of EFRDEGs in EM, a network of protein-protein interactions (PPI) was set up utilizing the STRING database, and a composite score of ≥0.15 was used as the output condition to facilitate the identification of crux genes for efferocytosis in EM. To investigate the molecular mechanism of efferocytosis in EM, the ChEA3 (https://amp.pharm.mssm.edu/ChEA3), a transcription factor (TF) enrichment analysis database that identifies TF-target gene interaction pairs, was utilized to identify between the target gene and efferocytosis regulation, a *p* < 0.05 was deemed statistically noteworthy and visualized using Cytoscape (V 3.9.1), which is a bioinformatics statistical tool for visualizing data results.

### 2.4 Selection of diagnostic biomarkers related to efferocytosis in endometriosis

#### 2.4.1 Logistic regression model

Univariate logistic regression represents a generalized linear regression analysis model applicable to the automated diagnosis of diseases. In our study, logistic regression employing two response variables denoting the Ectopic and Eutopic samples was employed. A 3-fold cross-validation (CV) approach was relevant to facilitate the grouping and cross-validation of logistic regression models. An initial application of multivariate stepwise logistic regression analysis was employed to eliminate factors deemed insignificant for the response variable. Receiver operating characteristic (ROC) analyses were conducted to identify EFRDEGs with elevated diagnostic values in EM. ROC for threshold selection and model comparison. The EM disease prediction model is constructed based on Univariate logistic regression analysis. The region under the ROC curve (AUC) > 0.5 proves that these tests or models have certain diagnostic values. Only those *p*-values <0.05 and AUC >0.8 were retained to analyze in the next step.

#### 2.4.2 Screening diagnostic biomarkers by machine learning models

Following that, the least absolute shrinkage and selection operator (LASSO) and support vector machine (SVM) regression models were employed to filter and determine diagnostic markers, establishing a predictive model for disease diagnosis (Gentleman et al., 2005). Utilizing the LASSO regression algorithm, the trajectory of each variable’s alteration was examined, and the *λ* value was established through 3-fold cross-validation. The selection of the *λ* value followed the principle of minimizing the mean square error of the Lasso model, with the “glmnet” R package employed to mitigate the risk of overfitting. Additionally, our study incorporated the SVM machine-learning technique, widely utilized for category or regression. To prevent overfitting, a recursive feature elimination (RFE) algorithm was applied to pick the relevant genes.

The candidate diagnostic markers of EM were identified based on the outcomes derived from LASSO and SVM analyses. Subsequently, multivariate stepwise logistic regression analysis was conducted on the potential diagnostic markers to further refine the selection of diagnostic biomarkers. Finally, for assessing the model’s predictive accuracy, GSE37837 and GSE6364 were utilized as test cohorts, and the ROC curve was generated with the assistance of the “pROC” R package.

### 2.5 Construction and evaluation of a nomogram

A nomogram is built based on multivariate logistic regression, according to the degree of contribution of each influencing factor to the dependent variable in the model (represented by the regression coefficient), integrating multiple predictors, assigning a score to each influencing factor, and then use a line segment with a scale to plot the graph according to a certain proportion. Nomograms transform complex multivariate logistic models into visual graphics, making the results of the prediction model more readable and facilitating patient evaluation. Hence, we created a nomogram model for diagnosing EM built on the outcomes of multivariate logistic regression, utilizing the “rms” R package. Decision curve analyses (DCAs) were generated to assess the clinical utility of the nomogram. Additionally, GSE37837 and GSE6364 were utilized as the validation set, and the ROC curve was generated to validate the model’s reliability. An AUC >0.5 is indicative of a well-modeled performance. For a more thorough assessment of the predictive capacity of the nomogram model, the calibration curve was generated using the Horslem test. This test compares the disparities between the forecasted values and the observed values, with a *p* > 0.05 indicating a well-fitting model.

### 2.6 Immune infiltration and correlation between diagnostic biomarkers and immune cells

The CIBERSORT algorithm (https://cibersort.stanford.edu/) was utilized to forecast and quantify the composition and abundance of 22 distinct types of immune cells. CIBERSORT is an online tool that predicts the abundance of 22 immune cell types using gene expression data (Wang et al., 2022). The gene expression matrix of the training test was uploaded to CIBERSORT and we measured the presence of immune cells in each sample and compared them between groups using the Wilcoxon rank-sum test (Newman et al., 2015). A histogram and Violin Plot depicting the layout of immune cell infiltrates in every sample were generated using the “ggplot2″ and “vioplot” R packages. The “Corrplot” R package was employed to illustrate the correlations among immune cells in ectopic endometrial samples. To probe the conjunction between diagnostic biomarkers and the levels of immune cell infiltration, we conducted a Spearman correlation analysis to appraise the association between immune infiltration abundance and the expression of diagnostic biomarkers (Liu et al., 2022) and visualized using the “corheatmap” R package. Then, a scatter plot with a significant correlation between the two was plotted using the “ggplot2” R package.

### 2.7 Single-cell RNA sequencing of immune infiltration in EM

To explore deeper into the immune microenvironment of EM, we dissected the Single-cell RNA Sequencing (scRNA-seq) dataset to examine the distinct expression profiles of immune cells in the ectopic endometria of EM patients. scRNA-seq affords a comprehensive insight into gene expression across diverse cell types within the same sample, aiding in the certification of crucial cellular subtypes. The GSE213216 matrices were retrieved from the GEO database (Fonseca et al., 2023), which contain 31 ectopic and 10 eutopic endometria samples, and GPL24676 (Illumina NovaSeq 6000, *H. sapiens*) was used as the sequencing platform. We integrated data from multiple samples and performed standardization, dimensionality reduction, and clustering to identify cell types or subpopulations in the data. Loads the “Seurat” R package for scRNA-seq data analysis and employs “RunTFIDF” and “`RunSVD” for data normalization. “Harmony” is used for integrating and reducing dimensions of data. The FindNeighbors (dims = 1:30), and FindClusters (resolution = 0.6) parameters were set and used to identify different immune cell clusters. Loads the Seurat object and performs various transformations on the RNA expression data, “tidyverse,” “dplyr,” and “patchwork” R packages for data manipulation and plotting. Uniform manifold approximation and projection (UMAP) clustering is performed, and clusters are visualized taking advantage of the “clustree” R package. This is useful for understanding the structure and relationships in the data.

### 2.8 Gene set enrichment analysis for the single gene and kyoto encyclopedia of genes and genomes analysis

Next, Gene Set Enrichment Analysis (GSEA) and KEGG pathway analysis were employed to unveil the potential biological functions of the diagnostic biomarkers (Subramanian et al., 2005). Apart from that, the gene set variation analysis (GSVA) algorithm was utilised to measure the GSVA score for the top 20 significantly gene set of the MSigDb Hallmark collection using the “GSVA” package. Samples were categorized into two groups per the median values of hub gene expression levels, comprising the high and the low expression groups. Genes were arranged in descending order based on log2FC, and the background gene sets were obtained from the Molecular Signatures Database (MSigDB, http://www.gsea-msigdb.org/gsea/msigdb) (Liberzon et al., 2015). All these analyses were performed using the “clusterProfiler,” “org.Hs.e.g.,db,” “DOSE,” and “enrichplot” R packages (Yu et al., 2012), and the screening condition was *p* < 0.05.

### 2.9 Potential drugs targeting the diagnostic biomarkers

Grounded on the diagnostic biomarkers of EM, the Comparative Toxicogenomics Database (CTD; https://ctdbase.org) was employed to anticipate potential drugs for EM therapy. The network demonstrating biomarker-compound pairs was visualized using Cytoscape.

### 2.10 Verification of the diagnostic biomarkers results

Immunohistochemistry (IHC) and real-time quantitative polymerase chain reaction (RT-qPCR) analysis in the present study was executed on 10 ectopic and eutopic endometrial samples from ovarian EM patients who underwent surgery, stored in wax blocks by the Pathology Department of the First Affiliated Hospital of Guangzhou University of Chinese Medicine (GZUCM). The participants, aged between 25 and 49 years, had no other significant illnesses and refrained from using any hormonal medications in the 3 months preceding the surgery. The project was affirmed by the Ethics Committee of The First Affiliated Hospital of GZUCM number: NO. K [2021]053. The pathological results of all samples were identified by two experienced pathologists at our hospital.

About 20 mg of tissue was scraped from the paraffin block using a sterilizing scalpel. The Tissue RNA Extraction Kit (G3640-100T, Servicebio, China) was then utilized to extract total RNA following the manufacturer’s instructions. Subsequently, the SweScript All-in-One RT SuperMix for qPCR (G3337-50, Servicebio, China) was employed to reversely transcribe the RNA into complementary DNA within the PCR instrument (ETC811, EASTWIN, China). The qPCR was performed using 2 × Universal Blue SYBR Green qPCR Master Mix (G3326-05, Servicebio, China), We used 2 × Universal Blue SYBR Green qPCR Master Mix (G3326-05, Servicebio, China) (Ma et al., 2022), and the program was executed with the following parameters: pre-denaturation at 95°C for 30 s, denaturation at 95°C for 15 s, annealing at 60°C for 30 s, and extension at 60°C for 30 s, for a total of 40 cycles. We selected GAPDH as the internal reference, and the relative mRNA expression levels were computed using the 2^−ΔΔCt^ method. The primer details can be found in Table 2.

**TABLE 2:** qPCR primers.

Gene	Primer	Sequence (5′-3′)	Size
GAPDH	Forward	GGA​AGC​TTG​TCA​TCA​ATG​GAA​ATC	168bp
	Reverse	TGA​TGA​CCC​TTT​TGG​CTC​CC	
C3	Forward	GTG​AGC​CAG​GAG​TGG​ACT​ATG​TG	131bp
	Reverse	CAT​ATA​AAG​CCC​GCA​AGC​AT	
ARG2	Forward	CTG​TTG​TCG​GGG​GAC​TAA​CCT​AT	138bp
	Reverse	CTG​TAG​TCT​TCG​CCT​CTT​CCT​CT	
GAS6	Forward	ACC​ATC​CAG​GAA​ACG​GTG​AAA​G	106bp
	Reverse	AGT​CCA​GGC​TGT​AGA​AGG​CGA​A	

For IHC staining, the paraffin sections were immersed in xylene for dewaxing, hydrated with an alcohol gradient, and repaired using EDTA solution in a microwave oven. To block nonspecific antibody binding, paraffin sections were incubated with normal goat serum for 90 min, and then the primary antibodies of GAS6 (Abcam, ab264098, 1:200), C3 (Abcam, ab200999, 1:200), and ARG2 (Affinity Biosciences, AF0738-200, 1:200) were added dropwise and incubated overnight at 4°C. The following day, after rewarming, the tissue sections were soaked in 3% hydrogen peroxide J for 8 min and then rinsed with PBS. After rewarming, the tissue sections were soaked in 3% hydrogen peroxide J for 8 min and then rinsed with PBS. The sections underwent incubation with secondary antibodies (1:200) at room temperature for 1 h, followed by staining with diaminobenzidine solution (DAB Sigma, United States). Subsequently, the sections were counterstained with hematoxylin. The stained tissue sections were visualized and captured using a 3D HISTECH digital slice scanner. Protein expressions within the colored tissue sections were dissected utilizing Image-Pro Plus 6.0 software.

### 2.11 Statistical analysis

All bioinformatics analyses were performed using R 4.2.1 software. ROC analyses were carried out using GraphPad Prism 9.0, with AUC values computed to appraise the forecast capability of each characteristic gene. Image-Pro Plus 6.0 (Media Cybernetics, United States) was utilized for the analysis and interpretation of IHC findings. Comparisons between the two groups concerning independent variables were conducted utilizing the Paired Mann-Whitney (SPSS 29.0), The Wilcoxon rank-sum test and Spearman correlation were employed to investigate the correlation between EFRDEGs and diagnostic biomarkers, as well as the infiltration standard of immune cells. The findings of the study with *p* < 0.05 were deliberated statistically significant.

## 3 Results

### 3.1 Identification of EFRDEGs in endometriosis

**FIGURE 1:**
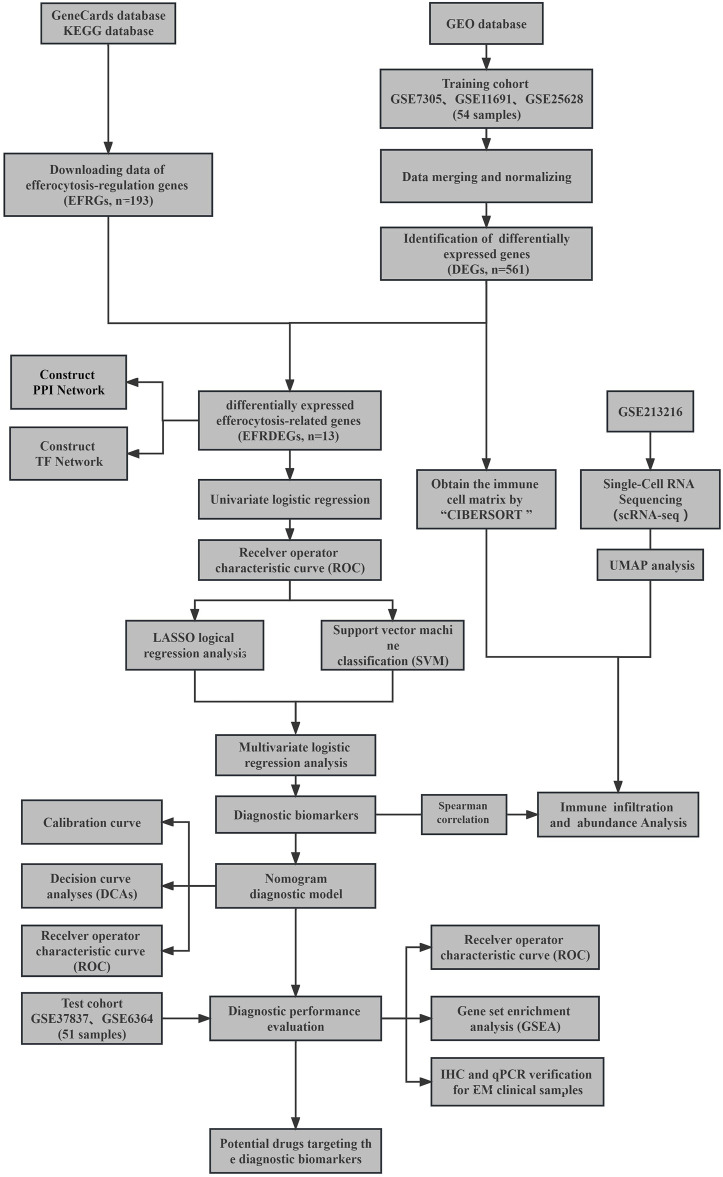
Flowchart depicting the stepwise screening strategy applied to bioinformatics data.

The analysis process of this study is shown in Figure 1.

**FIGURE 2:**
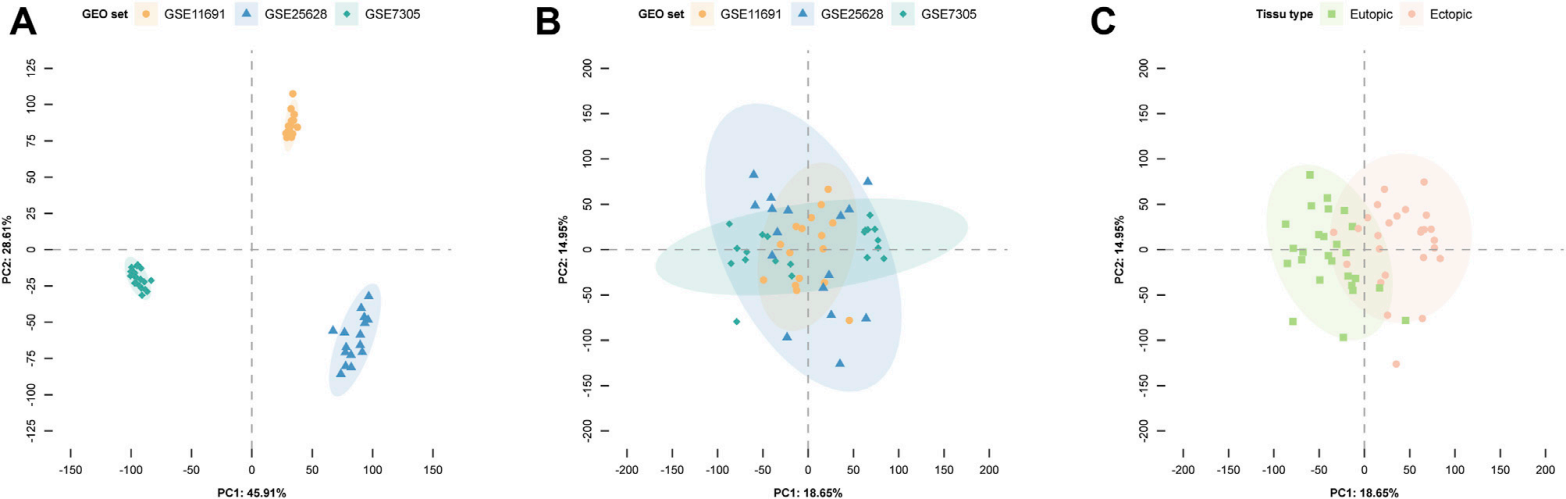
Principal component analysis (PCA) illustrates gene expression patterns across datasets. In the scatter plots, each point represents a sample based on the top two principal components (PC1 and PC2) of gene expression profiles. **(A)** The batch effect is obvious. **(B)** The removal of batch effect. **(C)** Batch effects are removed for ectopic and eutopic samples after correction. Colors represent corresponding samples across three distinct datasets.

The PCA results are shown in Figure 2, with A, B, and C representing different batch effects, demonstrating substantial removal of batch effects post-correction. A clear distinction was observed between samples from patients with EM and the control group (Figure 2C), indicating the suitability of the expression matrix for subsequent analysis.

**FIGURE 3:**
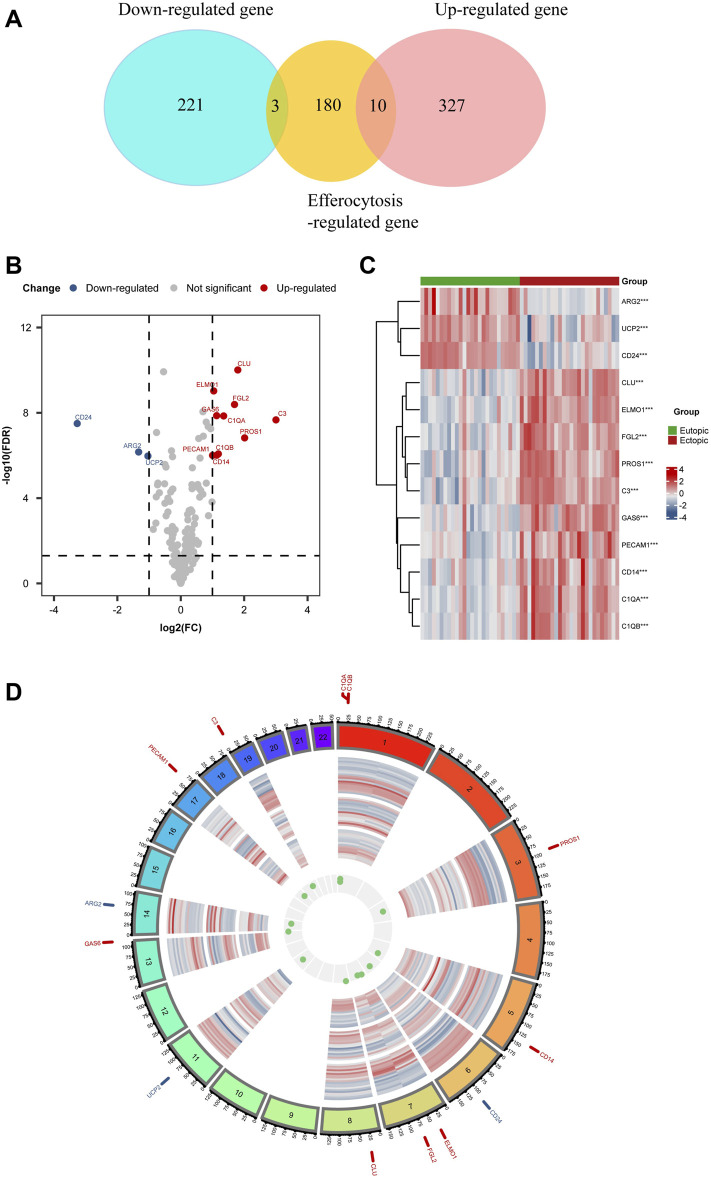
EFRDEGs in endometriosis: **(A)** Venn diagram illustrating the overlapping genes among DEGs, Efferocytosis-related genes, and EFRDEGs in EMs. In the representation, red demonstrates differentially upregulated genes, blue signifies differentially downregulated genes, and yellow represents efferocytosis-related genes. **(B)** Volcano plot displaying the 13 identified EFRDEGs in EMs. Red displays upregulated genes while blue displays downregulated genes. **(C)** Heatmap visualizing the expression levels of the 13 EFRDEGs. Red shows high expression, and blue shows low expression. **(D)** Chromosome region of the 13 ERDEGs. Gene names shown in red represent upregulated genes in the disease group while blue represents downregulated genes.

A thorough set of 141 EFRGs was sourced from the GeneCards databases, and an additional 150 EFRGs were retrieved from the KEGG databases (https://www.genome.jp/entry/pathway+hsa04148). After removing duplicates, a total of 193 EFRG genes were obtained. (Supplementary Material S1 provides detailed gene information). The clustered heatmap of DEGs unveiled the identification of a total of 561 DEGs from datasets related to endometriosis-related genes. We overlapped EFRGs with the DEGs and 13 EFRDEGs were discovered utilizing the “VennDiagram” R package, as depicted in a Venn diagram (Figure 3A), namely, Growth Arrest Specific 6 (Gas6), Complement C3 (C3), Clusterin (CLU), Protein S (PROS1), Fibrinogen Like 2 (FGL2), CD14 Molecule (CD14), Engulfment And Cell Motility 1 (ELMO1), Complement C1g A Chain (C1QA), Platelet And Endothelial CellAdhesion Molecule 1 (PECAM1), Complement C1g B Chain (C1QB), CD24 Molecule (CD24), Arginase 2 (ARG2), and Uncoupling Protein 2 (UCP2). The 13 EFRDEGs were visualized using a volcano map (Figure 3B) and heatmap (Figure 3C), of which Gas6, C3, CLU, PROS1, FGL2, CD14, ELMO1, C1QA, PECAM1, and C1QB were upregulated and CD24, ARG2, and UCP2 were downregulated. Furthermore, Figure 3D displays the Chromosome region of these EFRDEGs using circus v.0.69 to facilitate the recognition and analysis of similarities and differences generated in genomic intercomparisons.

### 3.2 Construction of PPI and TF networks for EFRDEGs

**FIGURE 4:**
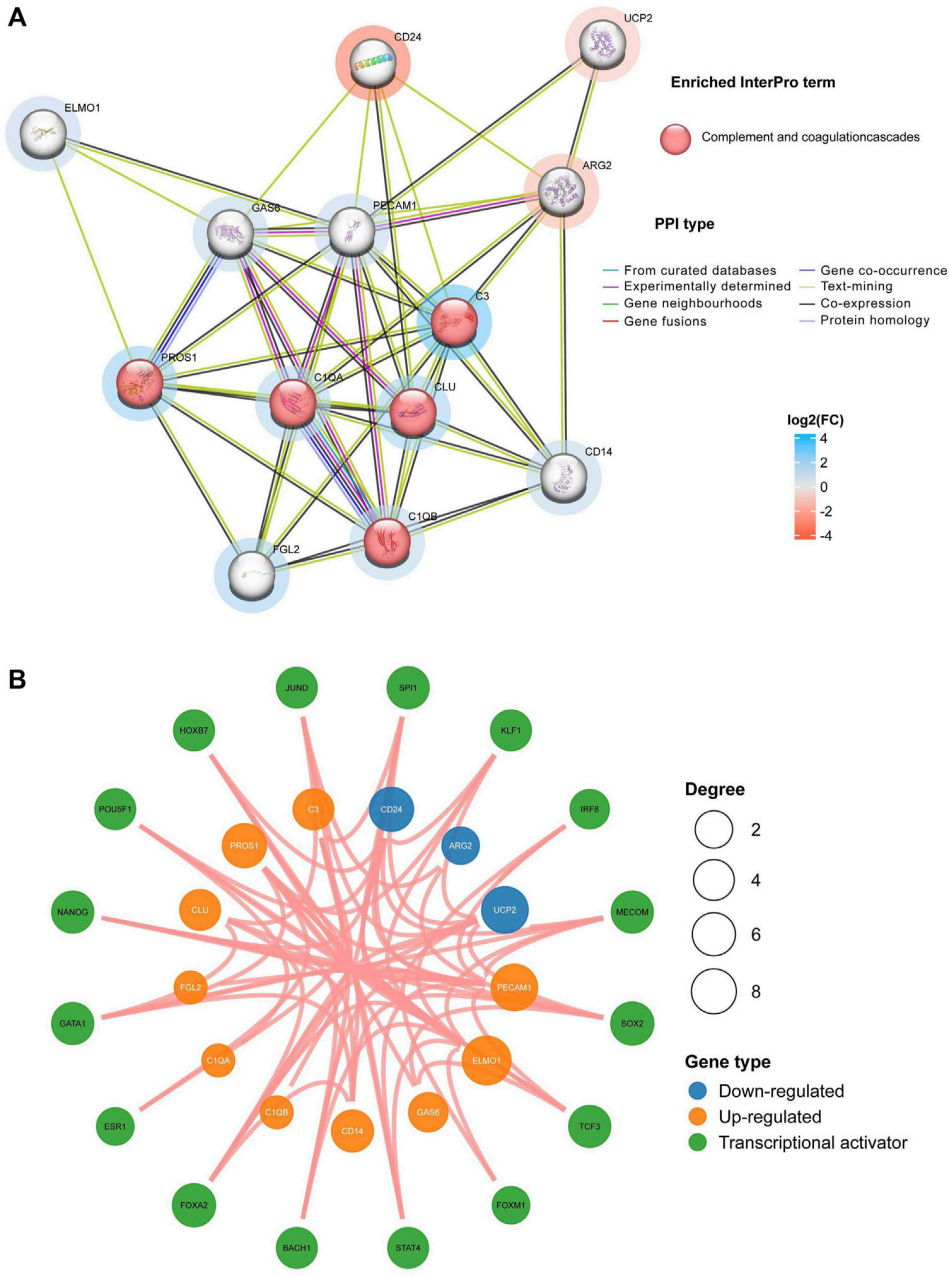
Construction of protein–protein interaction (PPI) and transcription factor (TF) for EFRDEGs: **(A)** Different nodes represent distinct proteins, and the color of the node represents the enriched pathway. The red node represents the enrichment of related proteins into the complement and coagulation cascade pathways. The color intensity around the node reflects the log2FC magnitude, with red representing downregulated EFRDEGs and blue representing upregulated. The color intensity around the node reflects the log2FC magnitude, with red representing downregulated EFRDEGs and blue representing upregulated EFRDEGs. **(B)** Green circles represent TF candidates predicted from the database. Only results with *p* < 0.05 were retained. Blue circles illustrate downregulated EFRDEGs, while orange circles illustrate upregulated EFRDEGs.

The interactions among these identified EFRDEGs were scrutinized through the PPI network, comprising 13 nodes and 47 edges, as depicted in Figure 4A. Highly connected proteins may have the same or similar functions, sorted nodes by degree, then, PECAM1, C3, C1QA, GAS6, C1QB, CLU, PROS1, ARG2, CD14, and FGL2 were selected according to the degree>5, which may be key factors affecting the entire metabolic or signal transduction pathway of efferocytosis defects related to EM. The analysis of the core molecule interaction network is shown in Figure 4B. In the TF-mRNA regulatory network, there are 16 TFs were found, and the yellow and blue circle represents EFRDEGs, red circle represents upstream transcription factors.

### 3.3 Machine learning-based selection of diagnostic biomarkers

**FIGURE 5:**
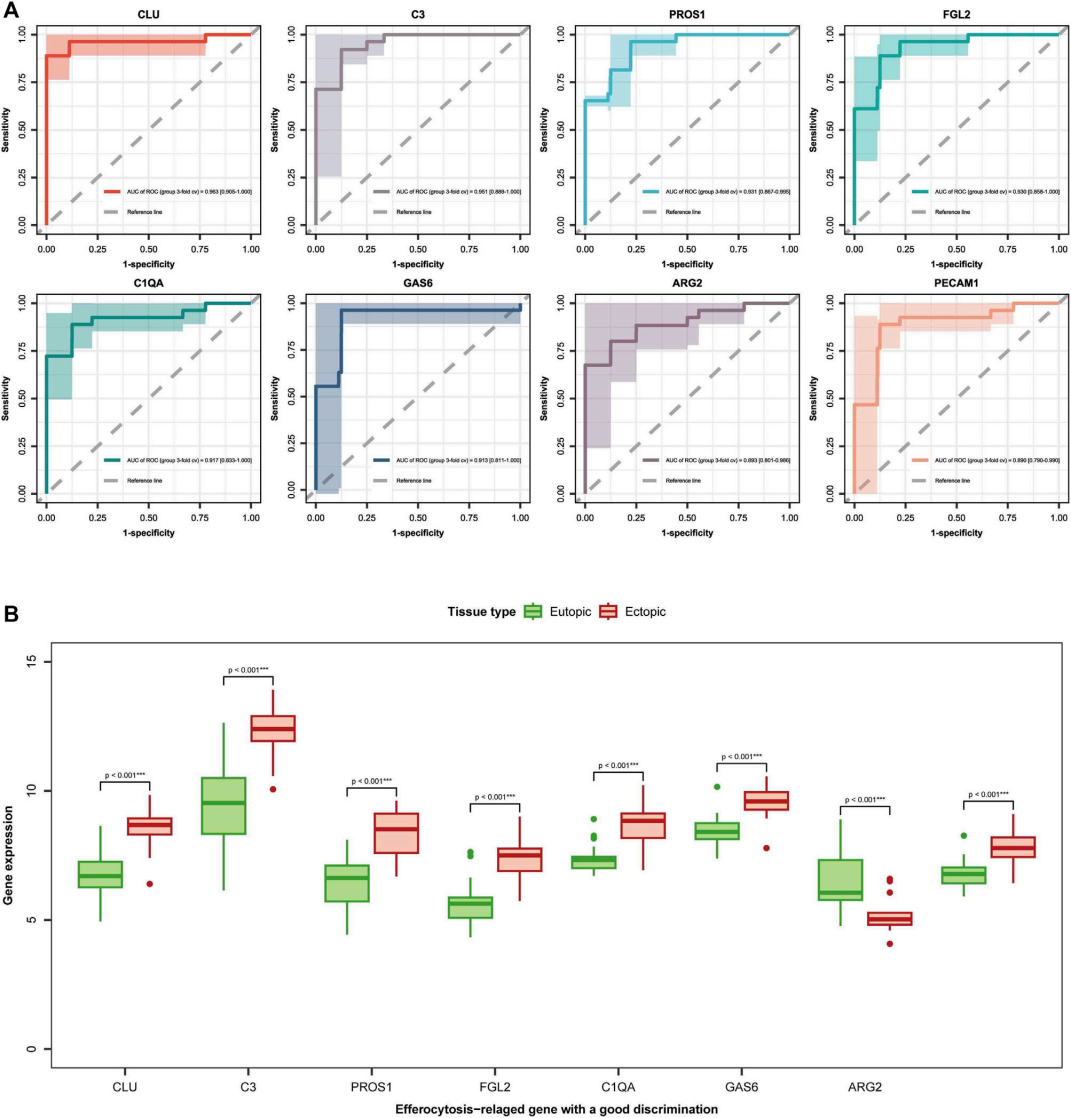
diagnostic markers selection: **(A)** Receiver operating characteristic (ROC) curves for the 8 EFRDEGs are displayed. The *x*-axis denotes the false-positive rate, while the *y*-axis represents the true-positive rate, quantified by sensitivity. The area under the ROC curve (AUC) measures the intensity of connection between the gene and the disease, with a higher AUC indicating a pretty association. **(B)** Box plots depict the expression levels of the eight chosen genes (CLU, C3, CLU, FGL2, PROS1, GAS6, C1QA, ARG2, and PECAM1) in both eutopic and ectopic endometrial tissues. Green represents eutopic endometria, while red represents ectopic endometria. ***p < 0.001 signifies a statistically great difference in gene expression between the two types of endometria.

To further understand the role of the 13 EFRDEGs in the diagnosis and prophecy of EM, we aim to identify hub genes from the 13 EFRDEGs for constructing a diagnostic prediction model. Initially, we conducted univariate analysis of the 13 EFRDEGs, the ROC curves for the 13 ERDEGs are shown in Figure 5A. With an AUC >0.8 as the cut-off value, eight genes were screened for further analysis. They are CLU, C3, CLU, FGL2, PROS1, GAS6, C1QA, ARG2, and PECAM1. Among them, CLU (AUC = 0.959%, 95%CI: 0.913–1.000) had the highest AUC value. A boxplot of the eight genes was plotted in Figure 5B.

**FIGURE 6:**
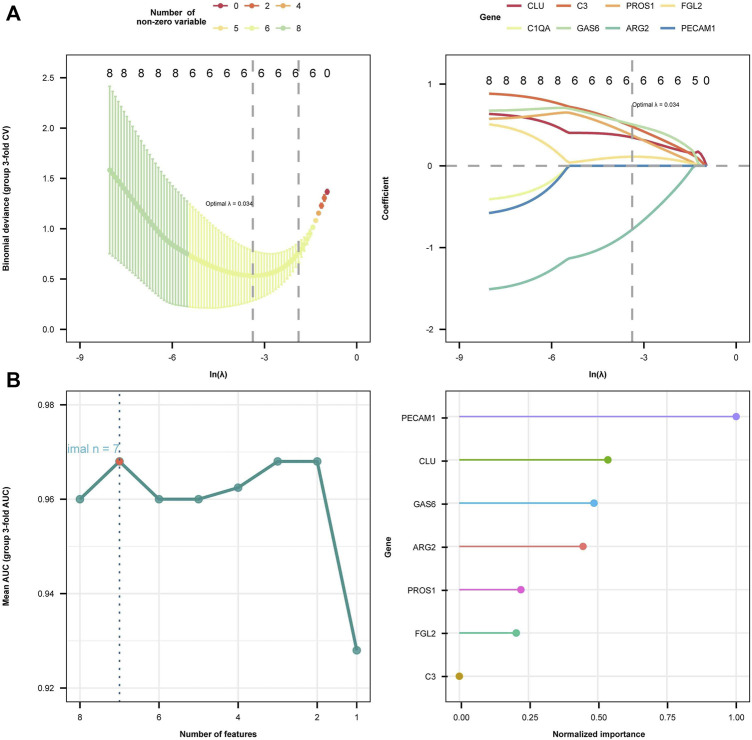
Diagnostic biomarkers selection using two machine learning methods. **(A)** The least absolute shrinkage and selection operator (LASSO) algorithm results are presented in two plots. In the left plot, the horizontal axis symbolizes log(λ) values and the vertical axis symbolizes regression cross-validation errors. The right plot displays the ln-transformed minimum log(λ) values along the horizontal axis and the corresponding coefficients on the vertical axis. Six genes whose coefficients were not 0 when lambda = 0.037 were screened out. **(B)** Support vector machine recursive feature elimination (SVM-RFE) regression model algorithm identified seven diagnostic biomarkers. The right plot illustrates the ranking of these seven feature genes according to their importance from highest to lowest as follows: PECAM1, GLU, GAS6, ARG2, PROS1, FGL2, and C3.

Subsequently, we employed LASSO regression analysis and SVM classification to further refine and recognize diagnostic markers. The outcomes from Lasso regression revealed that the six genes (ARG2, GAS6, C3, PROS1, CLU, and FGL2) had an ideal fit whose coefficients were not 0 when *λ* = 0.037 (the optimal sparsity parameter *λ* was 0.037, Figure 6A). The SVM classification algorithm recognized that seven genes (ARG2, GAS6, C3, PROS1, CLU, FGL2, and PECAM1) had significant classification effects (Figure 6B). The results of that intersection of LASSO regression analysis and SVM classification indicated that ARG2, GAS6, C3, PROS1, CLU, and FGL2 are potential markers for the diagnosis of EM. Ultimately, multivariate stepwise logistic regression analysis was performed for the six genes, and three genes (C3, GAS6, and ARG2) were screened, the *p*-values of the C3 were less than 0.05, result shown in Table 3.

**TABLE 3:** Multivariate stepwise logistic regression.

Hub gene	β	SE	Wald	OR	95% CI	P
C3*	1.391	0.566	2.459	4.019	[1.665–16.851]	0.0139
ARG2	−1.348	0.880	−1.532	0.260	[0.033–1.157]	0.1255
GAS6	1.056	0.903	1.170	2.875	[0.464–19.732]	0.2421

*p < 0.05, the result has statistical significance. Illustration: AIC, is the basis for stepwise regression screening, in the training test, the multi-factor AUC, was greater than 0.7 and at least one gene was significant, which indicates the results are meaningful.

### 3.4 Establishment and evaluation of diagnostic prediction model

**FIGURE 7:**
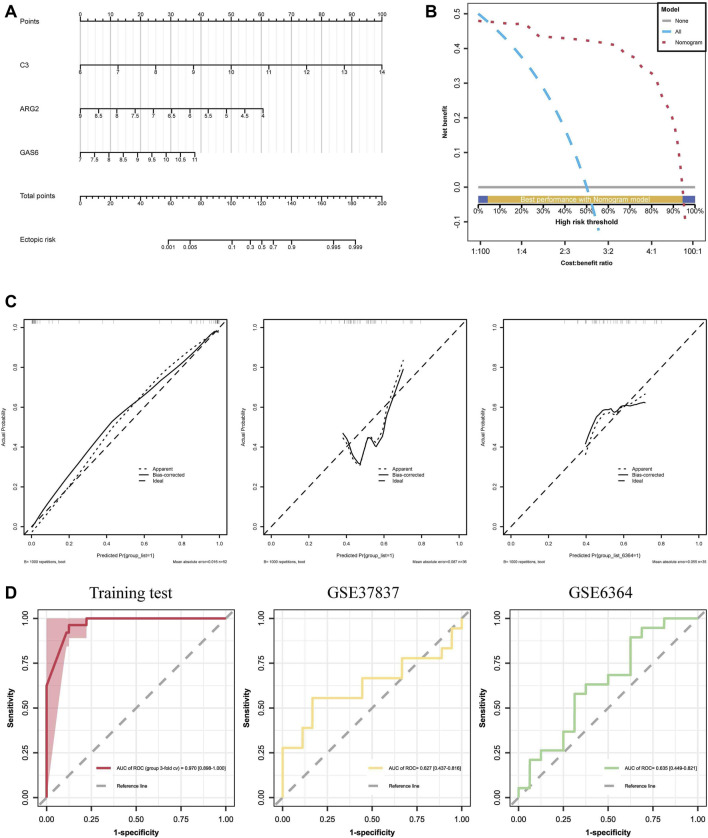
Establishment and assessment of diagnostic prediction model: **(A)** A nomogram of diagnostic biomarkers, where “Point” represents individual scores on the scale; ARG2, GAS6, and C3 correspond to the scores of each gene; “Total Point” represents the combined score of the three hub genes. **(B)** Decision curve analyses (DCAs) for the nomogram, show that the model curves are above the high-risk threshold curve. **(C)** Calibration curves of the hub genes, demonstrating good calibration of the combined model after bias correction. **(D)** ROC curve of the nomogram model with an AUC of 0.978, and the test sets GSE37837 and GSE6364, with an AUC of 0.627 and 0.635, respectively.

Using the training cohort, a nomogram model for the diagnosis and prediction of EM was constructed based on the C3, GAS6, and ARG2 genes (Figure 7A). Each predictor in the nomogram corresponds to a specific score, and the “total score” is the cumulative sum of the scores from the aforementioned predictors, and we can predict the risk of suffering from EM based on the “total points”. The DCA curves (Figure 7B) determined by 3-fold cross-validation showed that the model curves are all above the high-risk threshold curves, and the valid intervals of the nomogram were 4.35%–94.31%, which suggests that our nomogram model exhibits high accuracy and can serve as a foundation for clinical decision-making. After 1000 samplings, the calibration curve in the training and test cohort (Figure 7C) was close to the reference line, and the *p*-value was 0.1014, which indicates that the combined model overfitting was minimized, and the discrepancy between the actual EM clusters risk and the predicted risk was very small. The AUC of the nomogram model was 0.970 (95% CI: 0.898–1.000), demonstrating high feasibility for the nomogram diagnosis model. ROC curves depicted the satisfactory capability of the three-gene prediction model, with an AUC value of 0.627 (95% CI: 0.437–0.816) in the GSE37837 dataset and 0.635 (95% CI: 0.449–0.821) in the GSE6374 dataset (Figure 7D). These results indicate the efficacy of our diagnostic model in distinguishing EM from normal individuals.

### 3.5 Immune infiltration analysis results

**FIGURE 8:**
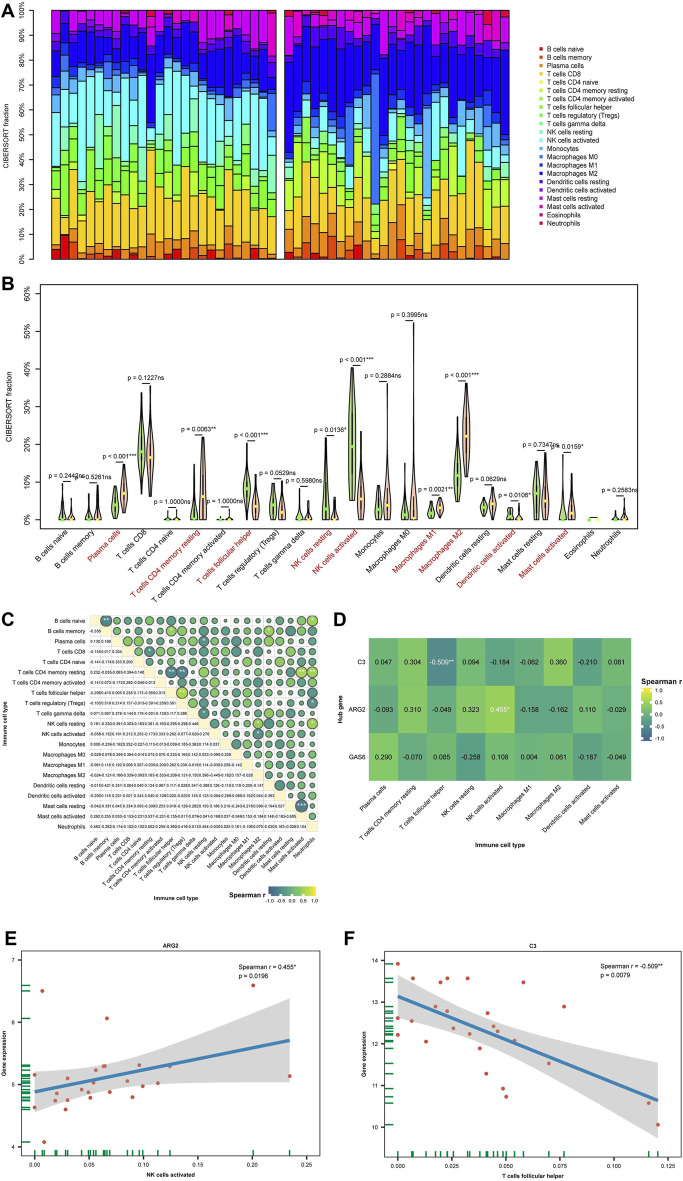
(Continued).

In this investigation, the CIBERSORT algorithm was employed to estimate the proportion of 22 immune cells in 26 eutopic and 26 ectopic endometrial samples, as illustrated in the histogram in Figure 8A. A violin plot in Figure 8B compares the immune cell infiltration in eutopic and ectopic endometrial samples. Compared with the eutopic endometria, the ectopic endometria showed a significant increase in the proportions of M2 macrophages (*p* < 0.001), plasma cells (*p* < 0.001), CD4^+^ memory T cells (*p* < 0.01), and dendritic cells (DC, *p* < 0.05). In contrast, the proportions of T follicular helper cells (Tfh, *p* < 0.001), activated natural killer (NK) cells (*p* < 0.001), resting NK cells (*p* < 0.05), and activated dendritic cells (DCs) (*p* < 0.05) were dramatically reduced. In Figure 8C, the conjunction between various types of immune cells are depicted, illustrating the close interconnection among these immune compositions, with CD4^+^ memory T cells notably associated with most immune compositions. Next, the correlation between diagnostic biomarkers and the infiltration levels of immune cells was further analyzed. The outcomes presented that hub genes correlate with the function of one or more cells in immune infiltration in Figure 8D, which indicates that the diagnostic biomarkers may have a significant impact on the immune microenvironment. Among them, there was a greatly negative correlation between C3 and Tfh cells, while ARG2 exhibited a significant positive correlation with NK cells (Figures 8E,F).

### 3.6 Single-cell RNA sequencing analysis of immune infiltration in endometriosis

**FIGURE 9:**
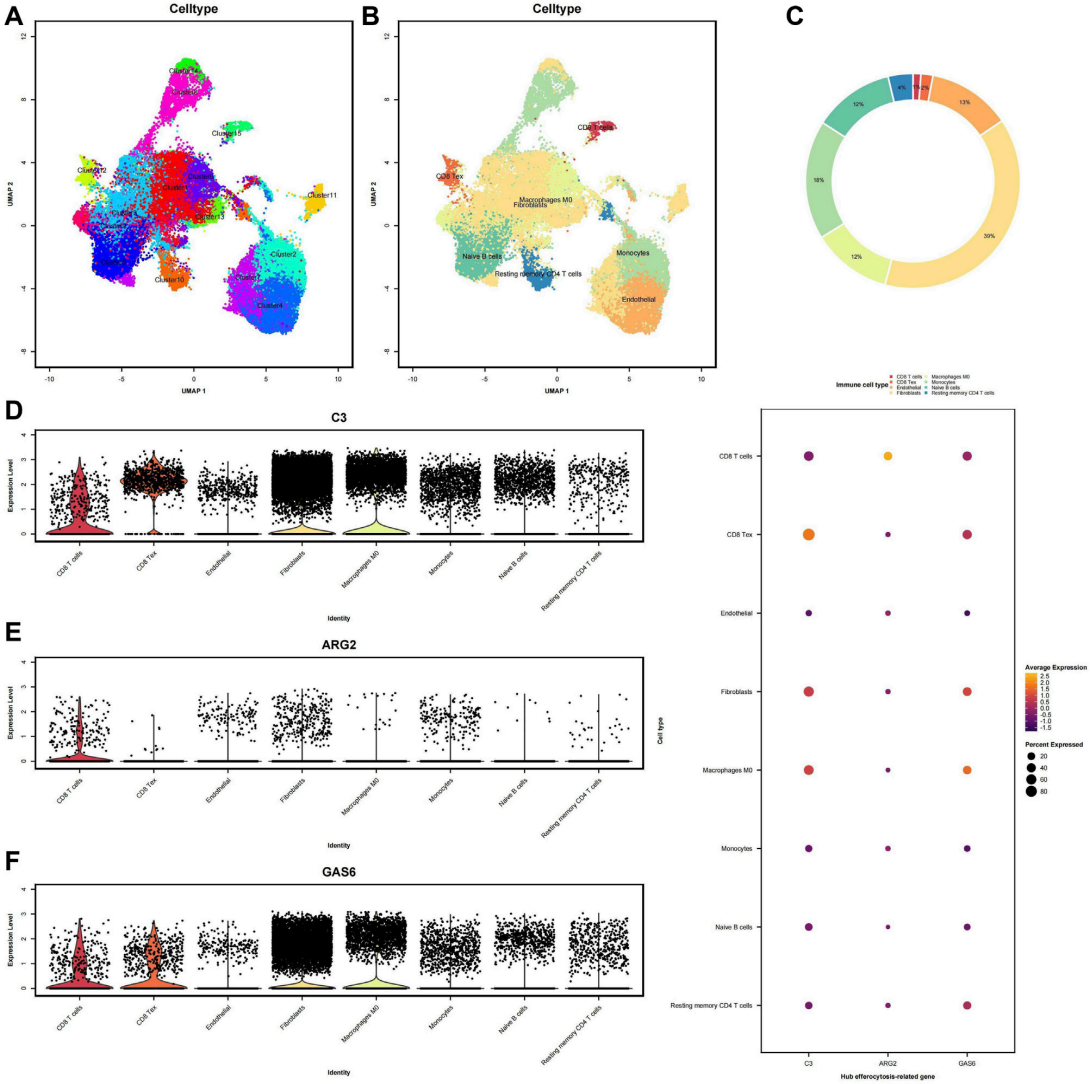
Single-cell RNA sequencing analysis (scRNA-seq) of immune infiltration: **(A)** Uniform manifold approximation and projection (UMAP) clustering plot showing a total of 15 distinct cell clusters. **(B)** Annotation of the main 8 immune infiltrating cell subtypes obtained from clustering. **(C)** Circular chart representing different cell clusters, with the values indicating the relative immune infiltration abundance. **(D–F)** Violin plots illustrate the immune infiltration abundance of the three diagnostic biomarkers. Each dot represents a single cell, with the *x*-axis indicating different cell clusters and the *y*-axis representing the expression levels.

To acquire a more abstruse comprehension of the variations in expression levels of diagnostic biomarkers across distinct immune cell populations, we opted for single-cell analysis in the GSE213216 cohort. We visualized the data and classified the cells into 15 cell subpopulations by UMAP (Figure 9A). Eight immune cell subpopulations were identified: macrophages, monocytes, endothelial cells, fibroblasts, CD8^+^ T cells, CD8^+^ Tex, naive B cells, and resting memory CD4^+^ T cells (Figure 9B). The relative immunological abundance of these 8 cell subpopulations is presented in the circular chart (Figure 9C), with fibroblasts being the most abundant, followed by monocytes. Then, We analyzed the diagnostic biomarkers in 8 cell subpopulations. The violin plot reflects the diagnostic biomarkers expression distribution of each cell in the subpopulation (Figures 9D–F). In the macrophages and fibroblasts subpopulation, C3 and GAS6 have higher expression levels, but are insensitive to low-expressed genes, for example, The distribution of ARG2 expression is unclear. The bubble chart can reflect the average expression level and cell proportion of hub genes in the subpopulation, at the same time, there is a certain detection rate for low-expression genes. C3 exhibits the highest expression level in CD8 Tex cells, whereas GAS6 demonstrates the highest average expression level in macrophages.

### 3.7 Functional enrichment analysis and potential drugs targeting for the diagnostic biomarkers

**FIGURE 10:**
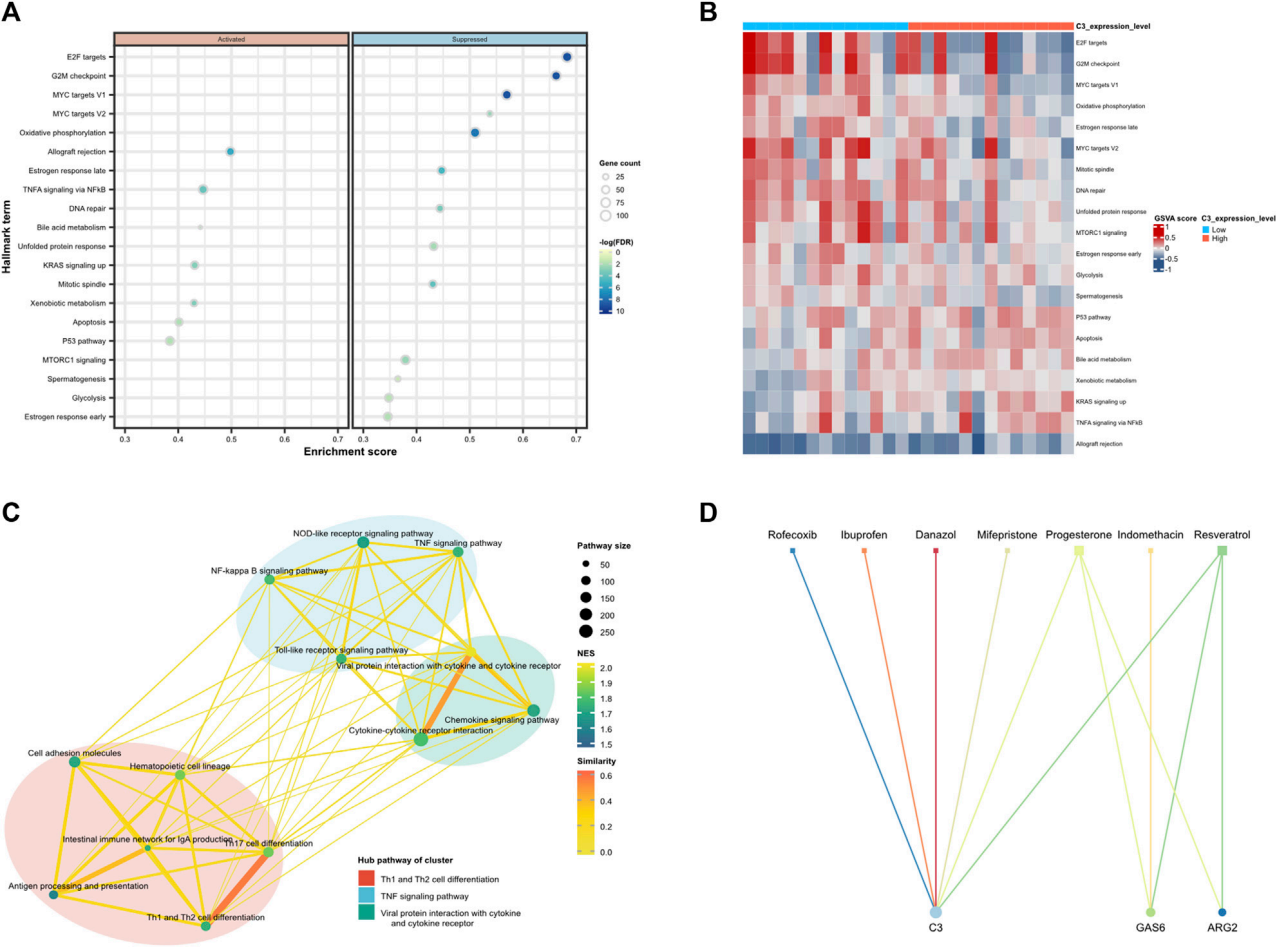
Functional enrichment analysis of C3 and potential drugs targeting diagnostic biomarkers. **(A)** Dot plot depicted the 20 most relevant Hall mark term with a *p*-value less than 0.05 ranked by gene ratio. Dot size is proportional to the number of overlapping genes. *P*-values are colour-coded according to the colour scale. **(B)** Heatmap plots of gene set variation analysis (GSVA) scores of the mSigDb Hallmark gene sets for the training set are shown for the TOP 20 sets with the highest significance in high-risk score level vs. low-risk score level comparison of C3. **(C)** Clustering network of significantly enriched KEGG pathways in the GSEA analysis, which deletes pathways related to disease types. The nodes represent the significant KEGG pathways and the edges represent the similarity between them and are coloured by normalised enrichment score (NES). The lines connected to similar pathways are coloured by similarity. **(D)** Protein-drug interaction network. Circle represent the hub dysregulated genes, while squares indicate the interacting drugs molecules. Node size is proportional to the degree (number of coincident edges).

Then, we performed GSEA analysis for genes with *p*-value < 0.05 in logistic bidirectional elimination regression to understand the potential biological roles. Figure 10A illustrates the bubble chart of GSEA of C3 has shown that genes exhibiting elevated expression were enriched in the allograft rejection, TNFA signaling via NFkB, and KRAS signaling up pathways, conversely, genes with reduced expression were notably enriched in pathways associated with E2F targets, G2M checkpoint, and MYC targets V1. Figure 10B illustrates the extent of pathway enrichment in individual samples using the “GSVA” R package. In high-level expression of the C3 group, the P53 pathway, Apoptosis, and TNFA signaling via NFkB pathways were significantly enriched. Subsequently, we downloaded the human “KEGGpathway” R data and excluded EM-related pathways. According to the KEGG categories, the outcomes of the C3 single-gene enrichment analysis were clustered using “clusterProfiler,” and “enrichplot” R package to find the commonality of the related pathways and calculated the mean and standard deviation of each cluster, *P* value<0.05 are statistically significant. As illustrated in Figure 10C, the central pathways within the cluster were Th1 and Th2 cell differentiation, TNF signaling, and viral protein interaction with cytokines and cytokine receptors.

Furthermore, with the application of the CTD chemicals database, we appraised potential therapeutic drugs for the therapy of EM by scrutinizing the diagnostic biomarkers we found. The result revealed that 7 drugs, namely, Ibuprofen, Danazol, Indomethacin, Mifepristone, Progesterone, Resveratrol, and Rofecoxib were filtered, among them, six drugs targeting C3, three drugs targeting GAS6, and two drugs targeting ARG2. Presumably, they can either reverse or induce the expression of hub genes, thereby influencing the state of EM, as depicted in Figure 10D.

### 3.8 RT-qPCR and immunohistochemistry analysis

**FIGURE 11:**
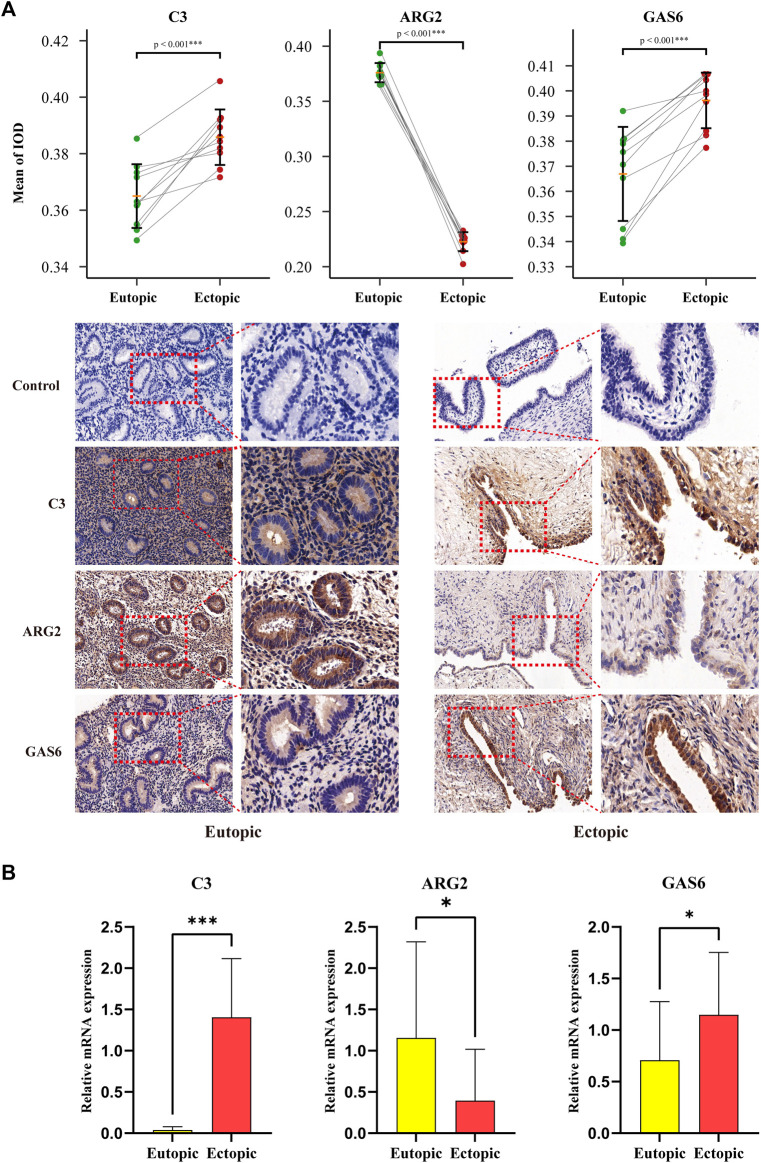
RT-qPCR and Immunohistochemical Analysis for diagnostic biomarkers. **(A)** Relative protein expressions of three diagnostic biomarkers in the ectopic and eutopic endometria, as determined by IHC techniques. **(B)** Relative expressions of three diagnostic biomarkers in the ectopic and eutopic endometria, as decided by qRT-PCR analysis. (*p < 0.05, **p < 0.01, ***p < 0.001).

qRT-PCR and IHC were carried out on 10 pairs of matched eutopic and ectopic endometrial tissue samples to verify the expression levels of three diagnostic biomarkers. IHC staining of EM tissues using anti-C3, anti-GAS6, and anti-ARG2 showed higher expressions of C3 and GAS6 and lower expressions of ARG2 in ectopic endometria relative to eutopic endometria, the difference was statistically significant (Figure 11A). Simultaneously, qRT-PCR was performed using total RNA extracted from 10 pairs of eutopic endometria and EM tissues. The results revealed higher expressions of C3 and GAS6, and lower expressions of ARG2 in ectopic endometria compared to eutopic endometria, and the difference was statistically significant (Figure 11B).

## 4 Discussion

EM is highly prevalent in Females in their reproductive years and represents an important cause of failure to conceive and preserve, decreased fertility, severe dysmenorrhea, and chronic pelvic pain; moreover, it increases the likelihood of developing epithelial ovarian cancer and cardiovascular disease (Mortlock et al., 2021; Fonseca et al., 2023). Surgery followed by postoperative pathological inspection is considered the gold standard for diagnosing EM, because the development of EM is usually slow, and the clinical diagnosis of EM is frequently delayed. The globally accepted definition of delayed diagnosis in EM is the duration between the initiation of pain signs or the emergence of endometriotic cysts and the surgical confirmation of EM (Ballard et al., 2006; Hudelist et al., 2012). A significant clinical challenge is the delay in diagnosis, not only does it result in the potential oversight of optimal treatment opportunities, but it also contributes to the progressive advancement of EM stages, heightened risks of infertility, and heightened surgical complexity and trauma. With the rapid upgrowth of bioinformatics technology and high-throughput sequencing, the integration of molecular biology and network science holds the promise of uncovering the origins of human diseases, potentially revolutionizing the diagnosis and treatment approaches for various medical conditions (Silverman et al., 2020). EM patients have an altered transcriptome, and variations in gene expression contribute to the non-surgical clinical diagnosis of EM has been emphasized. An expanding body of research indicates that the immune response significantly contributes to the pelvic microenvironment in EM. The proliferation of endometrial cells, enhancement of invasion, and local angiogenesis of the ectopic endometria are associated with alterations in the local immune microenvironment. In recent years, more and more researchers have been looking for novel EM diagnostic biomarkers and investigating the constituents of immune cell infiltrates in EM, which could potentially positively influence the clinical of patients with EM. Akter et al., 2019 evaluated the performance of different supervised machine learning methods in distinguishing ectopic endometria from EM patients and control endometrial samples, utilizing both transcriptomics and methylomics data. The study illustrated that machine learning methods utilizing transcriptomics or methylomics data provide a dependable channel for classifying EM. Moreover, numerous bioinformatics investigations on EM have revealed a close association between various immune cell subtypes and the biological processes of EM. These studies have also identified diverse biomarker genes implicated in EM, such as CCR5, MRC1, SYK, NOTCH3, SNAPC2, and PTOV1(Akter et al., 2019; Wang et al., 2022). Xie et al., 2022 suggested that the overexpression levels of DZWINT and AQP1 on endometriotic tissue mediated underlying immune system-associated pathology between M2 macrophages, activated mast cells, memory B cells, and activated NK cells, etc.

We employed CIBERSORT to assess the composition of immune cell infiltrates in matched eutopic and ectopic endometrial samples. The findings point to a latent association between the pathogenesis of EM and an elevated presence of M2 macrophages, plasma cells, CD4^+^ memory T cells, and activated dendritic cells, along with reduced infiltration of T follicular helper cells, NK cells, and activated mast cells. Studies conducted earlier have indicated the pivotal role of macrophages in the onset and progression of EM (Vallvé-Juanico et al., 2019). In EM samples, there is a heightened presence and activation of macrophages, primarily attributed to their phagocytic activity in clearing red blood cells and damaged tissue fragments. Research indicates that the pro-inflammatory immune population becomes activated in the early stages of EM development, leading to the prevalence of M1 macrophages trying to clear the ectopic endometria. (Mei et al., 2022). As the disease progressed, the proportion of M1 macrophages gradually decreased and the proportion of M2 macrophages increased. This shift is more pronounced in later stages (stages I–IV), thereby creating an immune microenvironment dominated by M2 macrophages, which promote local vascular growth and adhesion formation, and inhibit the clearance of endometriotic cells by other immune cells (Rakhila et al., 2016; Miller et al., 2020). Up to now, the specific characteristics of macrophages in the peritoneal fluid of EM patients remain a subject of debate (Guo et al., 2020). In the present study, the analysis of immune infiltration illustrated an increased presence of both M1 and M2 macrophages in ectopic endometrial tissue competed with eutopic endometrial tissue, with a predominant elevation in M2, suggesting a more significant involvement of M2 macrophages in the pathogenesis of EM. Studies have shown that in peritoneal fluid, in addition to macrophages, which represent the largest immune population, T cells and DCs also dominate (Guo et al., 2020). Our findings indicated an elevation in CD4^+^ resting memory T cells and DCs in ectopic endometrial tissue, suggesting the initiation of a prolonged immune response in individuals with heterotopic disease. Increased DCs possess potent antigen-presenting capabilities and are indispensable for T-cell activation (Lai et al., 2022). Furthermore, we found that Tfh cells are increased in eutopic endometria. Tfh cells, a CD4^+^ T-cell subset, are crucial for B-cell differentiation, memory B-cell generation, and high-affinity antibody production. The abnormal number or function of Tfh plays a crucial role in the development of autoimmune disorders such as rheumatoid arthritis (RA) and systemic lupus erythematosus (SLE). (Qi et al., 2023). This may indicate that EM may be closely related to autoimmune diseases. Besides, the analysis of immune infiltration indicated that the proportion of both activated, and NK cells was reduced in ectopic lesions. NK cells are essential to the innate immune system and denote a type of lymphocyte that clears ectopic endometrial cells. Dysfunction or diminished cytotoxicity of NK cells can lead to the development of EM (Lai et al., 2022). This was also sustained by Drury et al., 2018, who showed that in contrast to normal endometria, a notable reduction in uterine NK cells was observed in ectopic endometria. This decline in NK cells may be associated with the persistence of ectopic endometrial cells, resulting in the early development of lesions. Delving deeper into the pathogenesis of EM from an immunological standpoint can provide valuable insights, potentially offering novel avenues for early, non-invasive diagnosis, and therapy of the condition.

Efferocytosis, a crucial mechanism within the immune system, is liable for clearing up apoptotic cells in the body, contributing substantially to the maintenance of internal environment homeostasis and influencing various physiological and pathological processes (Doran et al., 2020). Apoptotic cells are recognized, engulfed, and digested by phagocytes to prevent undergoing further necrosis and releasing substantial amounts of inflammatory mediators. Efferocytosis can be carried out by professional phagocytic cells, such as macrophages and DCs, and on a smaller scale by non-professional phagocytic cells, such as endothelial cells (Siamon and Annette, 2018). Highly efficient efferocytosis within the organism is a fundamental element in preserving immune system homeostasis. Studies have shown that the role of efferocytosis in atherosclerosis is only approximately 1/20th of the physiological levels for a variety of reasons, such as macrophage recognition of apoptotic cells or macrophage dysfunction (Liao et al., 2018). Furthermore, studies have demonstrated that malignant cells can exploit the efferocytosis process to present molecular signals on their cell membranes, evading immune detection and surveillance by altering macrophage polarization and abundance (Werfel and Cook, 2018). There is currently a lack of research on efferocytosis in EM, the research of efferocytosis may conduce to the further understanding of the immune system’s role in the pathogenesis of EM. Therefore, investigating the correlation between efferocytosis and EM could uncover novel markers for diagnosing EM and identify fresh targets for treatment.

In our research, we associated the DEGs in the GSE7305, GSE11691, and GSE25628 datasets with genes engaged in the development of efferocytosis, and a cumulative 13 EFRDEGs were found in ectopic and eutopic endometria samples. Then, a univariate analysis was employed to pinpoint eight genes with close associations to EM. Among these genes, six hub genes (ARG2, GAS6, C3, PROS1, CLU, and FGL2) were further filtered and recognized as diagnostic biomarkers through the implementation of LASSO regression and SVM-RFE algorithms. Subsequently, multivariate stepwise logistic regression analysis and a nomogram model were employed for constructing a diagnostic prediction model comprising three genes (C3, GAS6, and ARG2). The ROC curve of the nomogram model with an AUC of 0.978, and the test sets GSE37837 and GSE6364, with an AUC of 0.627 and 0.635. The calibration and decision curves demonstrate that the model exhibits excellent predictive capability. Furthermore, in our study, we delved into the analysis of correlations between the three diagnostic markers and various immune cell infiltrations. Eventually, we validated the expression levels of three diagnostic biomarkers in our investigation of ectopic and eutopic endometrial issues from EM patients, and the outcomes were in concordance with the bioinformatics analysis. In contrast to the control group, the ectopic endometria group exhibited upregulated expressions of C3 and GAS6, along with downregulated expressions of ARG2.

Growth Arrest Specific 6 (GAS6) is a protein-coding gene, which was early explored to be upregulated in growth-arrested fibroblasts. This gene is commonly upregulated in miscellaneous cancers and has been attached to an unfavorable prognostic outlook (Ammoun et al., 2014; Chiu et al., 2015; Mullen et al., 2022). Increased protein levels are also linked to several health conditions, such as systemic lupus erythematosus, venous thromboembolic disease, and chronic renal failure (Lee et al., 2012; Kim et al., 2013; Schnegg-Kaufmann et al., 2019). Research has shown that Gas6 is expressed in host stromal cells, including fibroblasts, macrophages, and DCs within the tumor microenvironment (Tanaka and Siemann, 2020). Apart from neoplastic cells, Gas6 is detected in both luminal and basal mammary epithelial cells throughout puberty and adulthood and plays a role in the procedure of bone formation (Shiozawa et al., 2010; Mills et al., 2018). It is thought that be Gas6/Axl signaling pathway mediates the crosstalk between tumor and immune cells, thereby contributing to immune suppression and evasion within the tumor microenvironment to facilitate the growth of tumors, survival, and metastasis (Wu et al., 2018). Zdżalik-Bielecka et al., 2021 proposed that activation of Axl by GAS6 prompted actin remodeling, which further promoted micropinocytosis and contributed to cancer-cell invasion. Sun et al., 2003 investigated Gas6 and its receptors Axl in normal, eutopic, and ectopic endometria and revealed the expression of Gas6 and Axl mRNA in all the samples. However, Gas6 and Axl mRNA levels were markedly elevated in the ectopic endometria than the normal endometria (*p* < 0.05). This suggests that Gas6 and Axl signaling is dysregulated in EM and potentially contributes to abnormal growth. This aligns with the findings from our bioinformatics analysis.

As the predominant element in the complement system, C3 and its protein hydrolysis derivatives (C3b and C3c) play a pivotal role in initiating the complement system’s activation (Geisbrecht et al., 2022). The complement system constitutes a vital immune mechanism engaged in eliminating ectopic endometrial tissues and inflammatory response in the abdominal cavity (Yu et al., 2021). Tao et al. (Ricklin et al., 2010) contrasted the gene expression profiles of C3 in the ectopic and eutopic endometria of EM patients and discovered that C3 mRNA expression was notably higher in the ectopic endometria. Sayegh et al., 1996 demonstrated that C3 expression in individuals with EM was significantly elevated compared to those without EM. C3b can attach to pathogens, labeling their absorption and degradation through C3b receptors on immune cells (Yu and Zhang, 2021). In addition to producing cytotoxic effects, C3 induces other effects on the immune response, including modulatory effects, inflammatory mediators, and immune adhesion. Liszewski et al., 2013 found that the T cell-expressed protease cathepsin L (CTSL) was responsible for processing C3 into biologically active C3a and C3b. T cells derived from patients with autoimmune arthritis exhibited elevated intracellular C3a generation, mTOR activity, and proinflammatory cytokine production. Notably, the pharmacological inhibition of intracellular CTSL activity reversed these effects, underscoring the crucial role of intracellular C3 activation in regulating T-cell activity. In our study, we discovered that C3 was significantly negatively associated with the Tfh cells, this suggests a role between C3 and T cells in the pathogenesis of EM, and the precise potential mechanism requires further investigation. Moreover, GSEA and KEGG analyses uncovered the involvement of C3 in modulating immune-related signaling pathways, such as Th1 and th2 cell differentiation and Allograft rejection. Arginase is an enzyme liable for converting L-arginine into L-ornithine and urea and plays a crucial role in regulating arginine metabolism outside the urea cycle and in suppressing the synthesis of nitric oxide (Caldwell et al., 2015). Arginase 2 (ARG2), one of the isozymes of arginase in mammals, is primarily located in the mitochondria and is highly expressed in the small intestine, prostate, kidney, and lactating mammary gland (Pandey et al., 2014). ARG2 regulates various cellular functions and processes, including senescence, apoptosis, autophagy, and inflammatory responses in an arginase activity-dependent or independent manner (Ming et al., 2012; Xiong et al., 2013; Xiong et al., 2014). Abnormal expression of ARG2 has been increasingly associated with various diseases, particularly cardiovascular diseases (Polis and Samson, 2018; Grzywa et al., 2020). Xiong et al., 2013 established in mouse studies that Arg-II promotes mitochondrial dysfunction leading to vascular smooth muscle cell senescence, and induction of apoptosis occurs through intricate positive crosstalk involving S6K1-JNK, ERK, and p53, leading to atherosclerotic vulnerability phenotypes. Yepuri et al., 2012 showed in their research that senescent endothelial cells exhibit heightened expression of ARG2 and adhesion molecules. Silencing ARG2 in senescent endothelial cells enhances endothelial function and reduces adhesion molecule expression. There is limited research on ARG2 in EM. In this investigation, we identified that ARG2 was decreased in ectopic endometria than in eutopic endometria, which demonstrated a notable positive correlation with the reduction of NK cells, therefore, we surmise that low expression of ARG2 may reduce the senescence, apoptosis, and adhesion of epithelial cells in ectopic endometria, allowing the lesions to migrate and persist in the abdominal cavity.

We were interested in the influence of immune cell infiltration in the microenvironment of EM. Therefore, we took the single-cell transcriptional sequencing dataset of Fonseca to compare the changes in expression levels of EFRDEGs among various immune cellular components between ectopic and endometria samples. Fonseca et al., 2023 profiled transcriptome sequencing of individual cells in eutopic and ectopic endometria from EM creating a cellular atlas of endometrial-type epithelial cells, stromal cells, and microenvironmental cell populations across tissue sites. They found that lymphatic endothelial cell enrichment and endothelial cell compartment reorganization have been linked to somatic ARID1A mutation in epithelial cells. In our study, the findings showed that M0 Macrophages, Fibroblasts, and CD8 Tex cells were evaluated as the cell populations exhibiting the most significant changes in C3 and GAS6 expression levels, indicating that they could be major genes that affect how well immune cells perform efferocytosis in EM. Hull et al., 2008 suggest that molecular interactions between fibroblasts and endothelial cells may be the basis for ectopic lesion formation in EM. α-SMA (ACTA2), one of the myofibroblast-associated transcripts, was present in human EM tissue microarray gene lists. Attracted to areas of inflammation, myofibroblasts deposit collagen and other extracellular matrix (ECM) proteins, which provide growing stromal and epithelial cell shape. Fibroblasts were the most abundant subpopulation of immune cells in the ectopic endometria in our findings as well. Recent research has revealed that fibroblasts are not only structural components of tissues and organs but also dynamic participants in immune processes. They can coordinate immune responses by influencing the signaling pathways of relevant cytokines and chemokines as well as affecting immune cell differentiation and movement (Correa-Gallegos et al., 2021). Ma et al., 2021 confirmed that fibroblasts in ectopic endometria revealed distinct genetic variances when compared to eutopic and normal endometrial samples. In addition, high expression of estrogen receptor-b (ERb) was found in fibroblasts of the ectopic endometria, which is believed to facilitate the growth of ectopic lesions by enhancing the proliferative activity of the ectopic endometria and reducing apoptotic signaling. In conclusion, efferocytosis defects influence the immune microenvironment within the peritoneal cavity and at the local EM lesion site, allowing for increased resistance to the non-apoptotic programmed cell death of ectopic endometrial cells, which may enable the cells to spread and survive in the peritoneal cavity and then implant to form an endometrial lesion.

By analyzing the dataset retrieved from the GEO database, we identified three efferocytosis-related immune biomarkers in EM, presenting potential targets for pharmacological intervention. We utilized the CTD database to predict potential therapeutics for EM. The relationship between diagnostic markers and drug pairs was depicted through a network visualization using Cytoscape software. Certain drugs identified in our study have already been employed in clinical settings, such as Ibuprofen and indomethacin, which belong to NSAIDs, and are currently considered the preferred medication for managing pain associated with EM (Chen et al., 2021). Currently known hormonal drug treatments for EM recommended in China include commonly prescribed progesterone formulations, oral contraceptives, and gonadotropin-releasing hormone agonists (GnRHa). These medications can suppress ovarian function and urge low estrogen levels, thereby achieving the purpose of treatment. Danazol and Mifepristone can also treat EM through different mechanisms, but they are not commonly used clinically at present. Resveratrol is a physical polyphenolic compound known for its antiproliferative and anti-inflammatory properties, the study showed that it has beneficial effects on EM via anti-inflammatory and anti-angiogenic pathways (Dull et al., 2019). It is necessary to determine the mechanisms of Resveratrol in EM in future clinical studies.

## 5 Conclusion

In this study, we obtained three diagnostic biomarkers from the perspective of efferocytosis in EM and constructed a diagnostic model by bioinformatics technology and machine learning method, Moreover, the identification of a relation between the three diagnostic markers and immune cells has inaugurated a novel field for the mechanism studies of EM. This provides a novel thinking for the diagnosis, obstruction, and therapy of EM and lays the foundation for subsequent investigations. The inadequacy of our work is that the sample size might have been inadequate, given the constraints of the screening criteria. Validation with an expanded sample size is necessary. The study only found the relationship between the three diagnostic biomarkers and immune cells, but the concrete regulatory mechanism is not elucidated. Therefore, further in-depth research is needed to explore how efferocytosis-related genes can modulate immune cell infiltration and provide a reference for the clinical management of EM.

## Data Availability

The raw data supporting the conclusion of this article will be made available by the authors, without undue reservation.
